# Effectiveness of Eccentric Overload Training in Basketball Players: A Systematic Review

**DOI:** 10.5114/jhk/167469

**Published:** 2023-07-15

**Authors:** Omar Younes-Egana, Juan Mielgo-Ayuso, Marko D. M. Stojanović, Stephen P. Bird, Julio Calleja-González

**Affiliations:** 1 Independent researcher, Zaragoza, Spain.; 2 Department of Health Sciences, University of Burgos, Burgos, Spain.; 3 Faculty of Sport and Physical Education, University of Novi Sad, Novi Sad, Serbia.; 4 School of Health and Medical Sciences, University of Southern Queensland, Ipswich, Australia.; 5 Faculty of Education and Sport, University of the Basque Country (UPV/EHU), Vitoria, Spain.

**Keywords:** team sports, performance, countermovement jump

## Abstract

Several studies have confirmed the efficacy of flywheel eccentric overload training in order to improve or increase muscle volume and strength, as well as several performance-related fitness attributes, but to date, there are no studies that have reviewed the effects of these training methods in basketball. Therefore, the present systematic review aimed to collect the updated information about the influence of flywheel training on performance of basketball players. A search in four electronic databases (PubMed, SCOPUS, Web of Science, and Google Scholar) was conducted up to November 20, 2022. Articles were selected as valid for review if: (i) they were an experimental trial published in English; (ii) participants were basketball players without illnesses or injuries, and (iii) a rotational inertial overload method was used as a performance-dependent variable. A total of 93 articles were found. After filtering procedures, only seven studies were considered in this systematic review. In the selected studies, 203 participants were included, 58 females and 145 males. Focusing on basketball related performance variables, all the studies included the countermovement jump (CMJ), while five included sprinting and a change of direction test (COD). The results showed an improvement in performance-related variables associated with basketball (sprint, CMJ, and COD) through the use of inertial methods. Improvements were reported in professional, semi-professional and amateur players as well as both male and female players. However, given the low number of studies, additional investigations on this topic are warranted before a “clear picture” can be drawn concerning the effects of flywheel eccentric overload training in basketball players.

## Introduction

Basketball is an intermittent, court-based team sport in which both aerobic and anaerobic energy systems are stressed during the game, with players performing repeated actions such as jumps, sprints, turns, accelerations, decelerations, and changes of direction (COD) (Montgomery et al., 2010), all of which place high eccentric loads on players. Knowing the relevance of these types of actions on the court, several studies have reported beneficial effects of strength training which leads to improvements in high-intensity repeated actions that affect performance (Arede et al., 2019; Freitas et al., 2019; Gonzalo-Skok et al., 2019).

Over the past 20 years, a considerable amount of research has examined the effectiveness of eccentric training in athletic performance and injury prevention (Douglas et al., 2017; Higbie et al., 1996; Roig et al., 2009; Suchomel et al., 2018). This type of training may be beneficial for gains across the strength continuum (strength/hypertrophy/power), as suggested by Suchomel et al. (2018). The ability of skeletal muscle to produce higher force with lower muscle activation and metabolic cost (Higbie et al., 1996), higher solicitation of type IIx fibers (Duchateau and Baudry, 2014) and an increase in both the cross-sectional area and the development of a stiffer muscle-tendon unit during eccentric compared to concentric actions is well established. In addition, eccentric training induces positive adaptations at both molecular and neuromuscular levels, with an observed increase in cytokine interleukin-6 followed by elicited satellite cell proliferation following exercise. This is believed to play an important role in adaptive response to training (Douglas et al., 2017). At the neuromuscular level, it appears that cortical excitability is enhanced during eccentric actions resulting in a greater brain area being involved, indeed efferent motor output is not only regulated by central descending pathways, but also modulated by inflow from Golgi organs and muscle spindles (Douglas et al., 2017).

Given the reported benefits of eccentric training, flywheel eccentric overload training has become a popular training modality in recent times (Maroto-Izquierdo et al., 2017; Tesch et al., 2017). While traditional strength training offers phase-specific eccentric overloading, training with iso-inertial resistance (flywheel training) produces higher resistance during the entire range of motion, enabling greater power output in both concentric and eccentric phases of movement (Berg and Tesch, 1994). Flywheel devices were originally designed by Berg and Tesch (1994) to counteract the deleterious effect of microgravity on skeletal muscle (Tesch et al., 2004a). From this point of view, novel hardware, i.e., the YoYoTM Leg Press configuration was tested and validated in 1996 (Tesch et al., 2005).

These types of devices that can promote eccentric overloads are based on a flywheel anchored to a support structure with a rope that acts at a distance from the axis of rotation (Berg and Tesch, 1994). During the concentric phase, the applied force unwinds the flywheel’s strap, which begins to rotate and store energy. Once the concentric action is completed, the strap is fully stretched and the subject must resist the pull of the strap performing an eccentric braking action (Sabido et al., 2020). Simply put, the rate at which the strap is re-wound is based on the rate at which it is unwound, offering resistance through the eccentric phase of the exercise.

This new training paradigm gained popularity at the beginning of the century and the effects of iso-inertial training using flywheel devices have been extensively investigated to determine its transfer to both athlete’s performance (Gonzalo-Skok et al., 2016; Suarez-Arrones et al., 2020; Tous et al., 2006) and the general population (Maroto-Izquierdo et al., 2017; Nuñez Sanchez and Sáez de Villarreal, 2017; Tesch et al., 2017). Previous studies report that training using flywheel devices induces gains in muscle mass (Tesch et al., 2004a), improves maximal strength (Maroto-Izquierdo et al., 2017; Núñez et al., 2016; Petré et al., 2018), improves both concentric and eccentric voluntary strength (Tesch et al., 2004a), vertical jump (CMJ) performance (neuromuscular) (Raya-González et al., 2021; Tous-Fajardo et al., 2016), running speed (de Hoyo et al., 2015b; Tous-Fajardo et al., 2016) and improves electromyographic activity compared to traditional methods (Norrbrand et al., 2010). In addition, flywheel devices are transportable, enable performing exercises in different planes of motion and consequently better mimick sport specific movement patterns (Tous-Fajardo et al., 2016).

Despite the reported benefits of iso-inertial training, to date it still does not have a reliable base of support for its application (Suchomel et al., 2018). While recent meta-analyses have examined the effectiveness of flywheel training in sports performance (Allen et al., 2021; Petré et al., 2018; Raya-González et al., 2021; Vicens-Bordas et al., 2018), Raya-González et al. (2021) are the only ones who focused exclusively on elite athletes’ performance. Both Raya-González et al. (2021) and Petré et al. (2017) support the use of flywheel inertial training over free weights, while Vicens-Bordas et al. (2018) note that there are no additional benefits between both training methods. Nevertheless, several studies suggest that this type of training should be carried out 1 to 2 times a week for 8–10 weeks, with a volume of 2–4 sets and 6–10 repetitions in order to achieve improvements in variables such as the CMJ, COD, and the sprint (Beato et al., 2021; de Hoyo et al., 2015a; Gonzalo-Skok et al., 2016a; Tous-Fajardo et al., 2016).

The most direct application to sports performance that iso-inertial training has had in team sports was in elite soccer (Keijzer et al., 2022; Tesch et al., 2017). Studies carried out with soccer players have suggested that this type of iso-inertial training can reduce the injury risk (de Hoyo et al., 2014; Suarez-Arrones et al., 2018) and optimize return to play especially after a hamstring injury (Suarez-Arrones et al., 2020). It has also been suggested that applied throughout an entire soccer season, iso-inertial training can promote positive effects on both body composition and injury prevention (Suarez-Arrones et al., 2018). Regarding specific performance variables, its effectiveness in team-based sports such as soccer has been proven above any other, concluding that this type of training enhances change of direction, sprint and vertical jump performance (Gonzalo-Skok et al., 2016a; Raya-González et al., 2021; Tous-Fajardo et al., 2016).

Although several studies highlight the effectiveness of this training method applied in soccer, very few have applied this methodology in basketball performance (Cabanillas et al., 2020; Gonzalo-Skok et al., 2022; Gual et al., 2016; Hernández-Davó et al., 2018; O Brien et al., 2020; Sánchez-Sánchez et al., 2019; Stojanović et al., 2021). Both sports share the characteristic of being a team sport, but beyond that, it is difficult to extrapolate the results from soccer to basketball.

Therefore, the primary aim of this systematic review was to provide strength and conditioning coaches, medical professionals and performance staff with a compendium of peer-reviewed research examining the effects of flywheel eccentric overload training on basketball players and associated performance variables, and in turn, promote the development of evidence-based guidelines for such training modalities.

## Methods

### Search Strategy

The Preferred Reporting Items for Systematic Review and Meta-Analyses (PRISMA) guidelines were used for the definition of the inclusion criteria (Liberati et al., 2009). A structured search was carried out in the following electronic databases: PubMed, WOS, SCOPUS, and Google Scholar. Search terms covered a range of Medical Subject Headings (MeSH) and the conjugation of Boolean terms (AND & OR). Free-text words for key concepts associated with both flywheel and basketball were used resulting in the following unique search equation: ((“basketball" [MeSH Terms]) OR ("basketball" [All Fields])) AND (“strength training” [All Fields]) AND ((“eccentric overload” [All Fields]) OR (“isoinertial” [All Fields]) OR (“flywheel” [All Fields])). These terms were used as they have traditionally been used to refer to this type of training methodology (Maroto-Izquierdo et al., 2017). The search was completed without being confined to any specific years, with results being included up to the 20^th^ of November 2022.

The terms searched were related to flywheel training and basketball. Additionally, no other terms were used to increase the power of the analysis. Through this equation, all relevant articles in the field were obtained. The reference sections of all identified articles were also examined by applying the snowball method (Greenhalgh and Peacock, 2005). All titles and abstracts from the search were cross-referenced to identify duplicates and any potential missing studies, and then screened for a subsequent full-text review. The search for published studies was independently performed by two authors, and any disagreements were resolved through discussion with co-authors until a consensus was established.

### Inclusion and Exclusion Criteria

Studies were appropriate for inclusion if they met the criteria obtained by applying the PICOS model (Methley et al., 2014). P (Population): “basketball players”, I (Intervention): “flywheel devices training methodology”, C (Comparison): “traditional strength training”, O (Outcome): “performance-related variables”, and S (Study type): “interventional studies”. Other criteria were: (i) the study was experimental and published in English; (ii) the study included basketball healthy players without any pathology or present injury; and (iii) the study used a performance-related variable and iso-inertial training method (either a rotational pulley or a conical pulley). Studies were excluded if (i) the study did not have the minimum requirements as described above; (ii) the study did not report the results appropriately (mean and standard deviation); and (iii) the study period was less than four weeks. The OLE scale was applied (Tables 1 and 2), as per Saaiq and Ashraf (2017).

**Table 1:** OLE Scale.

Level	Evidence
*Level 1*	Meta-analysis of high-quality randomized controlled trials (RCTs) or RCTs
*Level 2*	Lower quality RCTs or prospective comparative studies
*Level 3*	Case studies or retrospective studies
*Level 4*	Cases without comparison of control groups
*Level 5*	Case reports or expert opinions

**Table 2:** Applied OLE Scale.

OLE	Nº Studies
L1 L2 L3 L4 L5	0 0 2 5 0

### Quality Assessment and Risk of Bias

To carefully consider the potential limitations of the included studies to obtain reliable conclusions, following the Cochrane Collaboration Guidelines (Higgins and Green, 2011), two authors independently assessed the methodological quality and risk of bias, whereas disagreements were resolved by third-party evaluation. Using the Cochrane Risk of Bias tool (Figure 2), the following items were included and divided into different domains: (1) selection bias (items, random sequence generation, allocation concealment), (2) performance bias (blinding of participants and personnel), (3) detection bias (blinding of outcome assessment), (4) attrition bias (incomplete outcome data), (5) reporting bias (selective reporting), and (6) other bias (other sources of bias). The assessment of the risk of bias was characterized as low risk (plausible bias unlikely to seriously alter the results), unclear risk (a plausible bias that raised some doubt about the results), or high risk (a plausible bias that seriously weakened confidence in the results).

Moreover, to determine the quality of the evidence, the authors reviewed the considered articles and provided PEDro (Physiotherapy Evidence Database) scores for each article. Only studies with PEDro scores of 5 or higher were considered for the systematic review. According to Moseley et al. (2020) the PEDro scale is an 11-item scale designed for rating the methodological quality of randomized control trials. Each satisfied item (except for item 1) contributes one point to the total PEDro score (0–10 points). The PEDro scores were extracted from the PEDro database.

## Results

### Main Search

**Figure 1:**
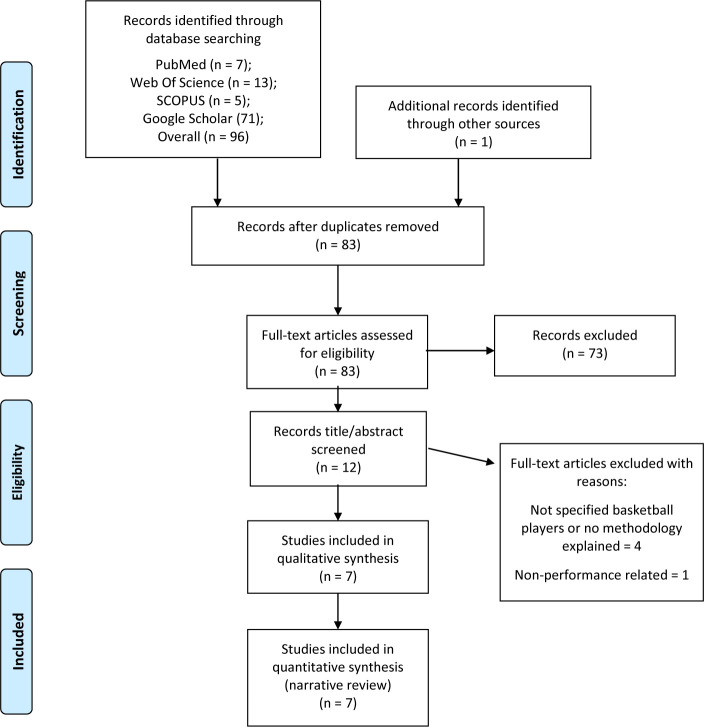
PRISMA flow diagram.

After the previous search terms were applied, the PRISMA graph was created (Figure 1) (Liberati et al., 2009). Once the specific filters were applied, the sample was reduced to a total of 7 studies valid for review (Cabanillas et al., 2020; Gonzalo-Skok et al., 2022; Gual et al., 2016; Hernández-Davó et al., 2018; O Brien et al., 2020; Sánchez-Sánchez et al., 2019; Stojanović et al., 2021). All of them included the previously established variables, that is, participants were male basketball players (Stojanović et al., 2021) or female basketball players (O Brien et al., 2020) and they analysed performance-dependent variables such as a vertical jump (CMJ) (Cabanillas et al., 2020), a sprint (Gonzalo-Skok et al., 2022) or a change of direction (COD) (Sánchez-Sánchez et al., 2019). To organize the data a Microsoft Excel spreadsheet was used, in which results were grouped arbitrarily (Table 3) according to players’ performance status (amateur or professional), the number of participants (n), study duration, and the methodology with the corresponding results obtained in the study.

**Table 3:** Breakdown of studies selected for systematic review

N	AUTHOR	YEAR	PARTICIPANTS - LEVEL	DESIGN	INTERVENTION	VARIABLES	OUTCOMES
**1**	Gual et al.	2016	81 basketball (B) and volleyball athletes, well trained	24 weeks; 2 groups. Both trained, but IG received additional YoYoTM training.	4 × 8 YoYoTM squat, 1 × week.	VISA-p, CMJ and squat power: CON and ECC	Squat-CON, Squat- ECC, and CMJ ↑ with YoYoTM VISA-R, VISA-L did not differ between groups
**2**	Hérnandez Davo et al.	2018	10 B amateurs	6 weeks; [flywheel bilateral support (n = 5) vs. flywheel unilateral support (n = 5)].	4 x 8 of bilateral or unilateral squat in flywheel device, 2 x week.	Power test in flywheel, CMJ, triple hop test and T-Test (COD)	T-Test and power test in flywheel ↑ in both groups Triple hop test may be greater for unilateral group.
**3**	Sánchez-Sánchez et al.	2019	24 B amateurs and soccer players	5 weeks; [HIT (n = 10) vs. CT (concurrent HIT eccentric overload training) (n = 12)]	Both: 2 HIT sessions; in addition, CT group performed EO (backwards lunges and hamstrings kicks with conical pulley and half-squats (kBox3) 2 × 6 and PI to 3 × 6 during; 2 x week	COD, RSA test, CMJ and 20-m shuttle run test	CT group ↑ COD, RSAbest, RSAmean, RSAslowest and CMJ compared to solely HIT.
**4**	Cabanillas et al.	2020	8 B professionals	8 weeks; EO (n = 4) vs. traditional squat (n = 4)].	PI in half squat; 4 × 10 to 6 × 10 reps (ProSquat ProInertial), 1 × week.	CMJ and 30-m sprint	EO ↑ CMJ and 30-m sprint
**5**	O Brien et al.	2020	20 B amateur females	4 weeks; [TET (n = 9) vs. FIT (n = 11)]	TET group: 4 x 8 65% (1RM) Back squat (2-s CON and 4-s ECC) FIT group: 4 x 10 (kBox 3); Both 2 x week	Back squat 1RM, CMJ, SJ, 10-m sprint, COD and S&R	1RM, 10 m and CMJ ↑ for FIT group
**6**	Stojanović et al.	2021	36 B players, well trained	8 weeks; [FST (n = 12) vs. TST (n = 12)] vs. CON (n = 12)	FST (D11 full device) and TST: One-arm dumbbell row, biceps curls, RD and HS (PI from 2 x 8 to 4 x 8) and rotational pall-of press (from 2 × (2 × 12−15) to 2 × (4 × 12−15); 1−2 x week	Lower limb isometric strength (ISOMET), 5 and 20-m sprint time, CMJ and T-test	ISOMET, CMJ and t-test ↑ FST than TST. ISOMET, CMJ, 5-m sprint and t-test ↑ FST than CON group.
**7**	O Gonzalo-Skok et al.	2022	24 elite youth B players	6 weeks; [VUL (n = 12) vs. VUH (n = 12)]	VUL group: Side-step, backward lunges, crossover cut & landing. VUH group: Lateral squat, shuffling step, lateral crossover & 90º lunge. 2 x week; Both PI from 1x6 to 1x10 with Versa Pulley.	CMJ, LJ & HJ, 5 and 20-m sprint time, modified 505 test, V-cut test & LSI	CMJ, HJ, LJ, LSI & 180º COD ↑ both groups. LJ ↑ VUL than VUH group

Legend: IG, intervention group; VISA-p, Victorian Institute of Sports Assessment patellar tendinopathy questionnaire; CON, concentric; ECC, eccentric; CMJ, countermovement jump; HIT, high-intensity training; EO, eccentric overload; PI, progressive increase; RSA, repeat sprint ability; TET, tempo eccentric training; FIT, flywheel inertial training; SJ, squat jump; COD, change of direction; S&R, sit and reach test; FST, flywheel strength training; TST, traditional strength training; CON, control; RD, Rumanian deadlift; HS, half-squat. VUL, variable unilateral training; VUH, variable unilateral horizontal; LJ; lateral jump; HJ, horizontal jump; LSI, limb symmetry index.

### Quality Assessment

**Figure 2:**
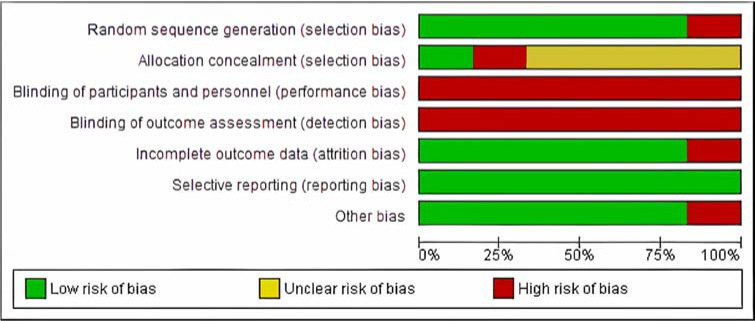
Risk of bias graph: review authors' judgements about each risk of bias item presented as percentages across all included studies.

The studies included in this systematic review showed a substantial amount of risk of bias concerning the blinding of participants and personnel and the allocation concealment, as well as several studies with incomplete outcome data (Figure 2). However, the seven studies obtained a high-quality methodology score (PEDro score ≥5/10), with a mean score of 6.5 according to the PEDro Scale.

### Target Study Variables

Table 3 shows all the characteristics of the seven studies finally included in the review (Cabanillas et al., 2020; Gonzalo-Skok et al., 2022; Gual et al., 2016; Hernández-Davó et al., 2018; O Brien et al., 2020; Sánchez-Sánchez et al., 2019; Stojanović et al., 2021), dating from the oldest (Gual et al., 2016) to the most recent one (Gonzalo-Skok et al., 2022).

The total of participants addressed in this review was 203, with 58 female and 145 male players. We found a male sex prevalence, except for the study of O Brien et al. (2020) standing out as the only one that used exclusively female players (n = 20) and Gual et al. (2016) using both female and male players.

Regarding the performance level of players, we differentiated among professionals (Gold Spanish basketball League) (Cabanillas et al., 2020), semi-professionals (Gonzalo-Skok et al., 2022; Stojanović et al., 2021), and amateur basketball players (Hernández-Davó et al., 2018; O Brien et al., 2020), differentiating between the latter to team sport players, whether it was basketball and volleyball (Gual et al., 2016) or basketball and soccer (Sanchez-Sanchez et al., 2019).

All qualified studies were experimental and with diverse duration, from 24 weeks ( Gual et al., 2016) to only four weeks during the preseason period (O Brien et al., 2020). The remaining studies were of 5-week (Sánchez-Sánchez et al., 2019), 6-week (Gonzalo-Skok et al., 2022; Hernández-Davó et al., 2018), and 8-week duration (Cabanillas et al., 2020; Stojanović et al., 2021).

All the studies used flywheel technology in their training methodology, either with a comparison to a traditional strength training group (Cabanillas et al., 2020; Gual et al., 2016), two flywheel group division (Gonzalo-Skok et al., 2022) or adding a control group (3 groups division) (Stojanović et al., 2021), with another type of eccentric training (O Brien et al., 2020) or with a group that used HIT as the main force expression (linear sprint) (Sánchez-Sánchez et al., 2019). Within the iso-inertial technology implemented, we could find two different machines, the rotational pulley, such as the commonly known “YoYoTM” (Tesch et al., 2004b) and their respective variants and versions; and the conical pulley that, as its name indicates, uses a cone as an inertial method (Sabido et al., 2020). Regarding the iso-inertial machine used in the selected studies, all the authors agreed on the use of the rotational pulley, highlighting the study by Sánchez-Sánchez et al. (2019), which used both the conical pulley and the rotational pulley, and the study conducted by Gonzalo-Skok et al. (2022) which only used the conical pulley.

Next, it was specified which exercise was the most common as a training method and at the same time, what volume was used by the different authors.

Regarding training volume, three studies included 4 sets of 8 repetitions (Gual et al., 2016; Hernández-Davó et al., 2018; Stojanović et al., 2021), one study included the volume of 6 sets of 10 repetitions (Cabanillas et al., 2020), with a lower volume established by Sánchez-Sánchez et al. (2019) of 3 sets of 6 repetitions, and with one study which included only one set, yet with the number of repetitions from 6 to 10 (Gonzalo-Skok et al., 2022). The trend was divided between not varying the number of sets and repetitions established throughout the entire intervention (Gual et al., 2016; Hernández-Davó et al., 2018; O Brien et al., 2020), and proposing a training volume at the beginning of the intervention and progressing towards a higher volume at the final training weeks (Cabanillas et al., 2020; Gonzalo-Skok et al., 2022; Sánchez-Sánchez et al., 2019; Stojanović et al., 2021). Another consensus was found regarding recovery time since almost all the interventions allowed 2 min of recovery between subsequent sets (Cabanillas et al., 2020; Gual et al., 2016; Hernández-Davó et al., 2018; O Brien et al., 2020; Sánchez-Sánchez et al., 2019; Stojanović et al., 2021).

Focusing on the performed exercises, we found that all of the studies implemented flywheel devices in lower limb training, except for Stojanović et al. (2021) who also included upper limb exercises, but in this case with free weights. Lower limb exercises were the squat, both bilateral (Gual et al., 2016; O Brien et al., 2020) and unilateral (Gonzalo-Skok et al., 2022; Hernández-Davó et al., 2018), the half-squat (Cabanillas et al., 2020), the Romanian deadlift (Stojanović et al., 2021), and hamstring kicks (Sánchez-Sánchez et al., 2019). All the exercises were executed once per week, also finding interventions up to twice per week (Sánchez-Sánchez et al., 2019; Stojanović et al., 2021).

As direct performance variables, the CMJ stands out, since it was applied in all the selected studies. The change of direction (COD) can also be emphasized, either using the T-test (Hernández-Davó et al., 2018; Stojanović et al., 2021), the Illinois test (Sánchez-Sánchez et al., 2019) or the 180º COD test plus the V-Cut test (Gonzalo-Skok et al., 2022). Only two studies did not use the COD as a performance-related variable, but did use the CMJ (Cabanillas et al., 2020; Gual et al., 2016). Another performance-related variable to highlight, used in almost all the interventions, was the sprint time evaluated at 10 m (O Brien et al., 2020), 20 m (Sánchez-Sánchez et al., 2019; Stojanović et al., 2021), 25 m (Gonzalo-Skok et al., 2022) or 30 m of the sprint (Cabanillas et al., 2020). Finally, a power test using the encoder of the iso-inertial device focusing on the squat (Gual et al., 2016; Hernández-Davó et al., 2018), a lateral and horizontal jump test (Gonzalo-Skok et al., 2022) and a flexibility test: “Sit and Reach” (O Brien et al., 2020), and tests such as the “VISA-P” questionnaire (Gual et al., 2016), which allows a clinical classification based on symptom severity, functional capacity and sports capacity (Hernandez-Sanchez et al., 2011), were also implemented.

It should be noted that no participant in all the proposed experimental studies had previous experience in eccentric resistance training. In the vast majority, familiarization sessions were applied to help players get acquainted with the established inertias (Cabanillas et al., 2020; Gual et al., 2016; Hernández-Davó et al., 2018; Stojanović et al., 2021). Different inertia values were applied, from an inertia of 0.025 kg∙m^2^ (Hernández-Davó et al., 2018) and 0.027 kg∙m^2^ (Gonzalo-Skok et al., 2022) to 0.11 kg∙m^2^ (Gual et al., 2016). Only Stojanović et al. (2020) and O Brien et al. (2020) agreed on a common inertia of 0.075 kg∙m^2^.

## Discussion

This systematic review aimed to provide strength and conditioning coaches, medical professionals and performance staff with a compendium of peer-reviewed research examining the effects of flywheel eccentric overload training on basketball players and associated performance variables. The results indicate that flywheel training applied 1–2 times a week led to improvements in performance variables such as the CMJ, the change of direction and the linear sprint (5–10 m). As described above, the results may be due to the neurological and physiological changes that eccentric training causes itself, or likely due to a combination of possible eccentric overload training and performance related exercises that were carried out.

Regarding the number of days of the intervention, there were more studies which proposed this type of training twice a week than only once (Gonzalo-Skok et al., 2022; Hernández-Davó et al., 2018; O Brien et al., 2020; Sánchez-Sánchez et al., 2019; Stojanović et al., 2021). Similar results were found in studies that implemented the same weekly volume of flywheel training in soccer players, also obtaining an improvement in variables such as sprinting and the CMJ (de Hoyo et al., 2014; Fiorilli et al., 2020). On the other hand, we also found studies in which performing this type of training once a week led to improvements in basketball players (Cabanillas et al., 2020; Gual et al., 2016), handball players (Sabido et al., 2017), and soccer players (Coratella et al., 2019; Tous-Fajardo et al., 2016), in the different performance-related variables considered by those authors, all having in common the improvement in the CMJ. Therefore, it cannot be conclusively stated that one approach is more effective than the other. More studies should be applied to provide relevant data regarding the frequency of flywheel training to improve performance.

One of the variables common to all the studies subjected to review was the CMJ (Cabanillas et al., 2020; Gonzalo-Skok et al., 2022; Gual et al., 2016; Hernández-Davó et al., 2018; O Brien et al., 2020; Sánchez-Sánchez et al., 2019; Stojanović et al., 2021). This action is frequently performed by basketball players as part of defensive (e.g., blocking and rebounding) and offensive (e.g., rebounding and shooting) manoeuvres during training and competition (Ben Abdelkrim et al., 2007; Ziv and Lidor, 2010). As Gual et al. (2016) state, this improvement in the vertical jump may be due to the lower limb power improvement, a variable that is also improved after adding a weekly flywheel training session in indoor sports such as basketball and volleyball. Following the same line, Cabanillas et al. (2020) stated that the CMJ improvement could be due to the specificity resulting from using the squat as a performed exercise on the flywheel device. That statement can be slightly altered since Stojanović et al. (2021) also reported improvements in CMJ performance in the group that followed flywheel training compared to traditional strength training, and the control group, training both the squat and the Romanian deadlift. On the contrary, in the study carried out by Gonzalo-Skok et al. (2022), the authors reported no benefits in bilateral vertical jump performance (ES = from 0.12 to 0.18) after 6 weeks of flywheel training, but they did report substantial improvements in the unilateral vertical jump performance (ES = from 0.35 to 0.62).

The addition of the Romanian deadlift (Stojanović et al., 2021) may even reinforce the hypothesis that training improvements are more closely related to flywheel training than to the chosen exercise, as long as it is aimed at the lower body. Recently, in the meta-analysis carried out by Maroto et al. (2017) exploring the effectiveness of training with iso-inertial devices, where the vertical jump was also examined, it was concluded that, as long as the jump modality used to assess jumping ability included an eccentric phase, it would be improved with flywheel training rather than with traditional training. Regarding what improvements this type of training has in different indoor sports such as handball, we also found that the vertical jump improved in participants who trained iso-inertially compared to those who trained traditionally, both the unilateral CMJ (Madruga-Parera et al., 2020) and the bilateral CMJ (Maroto-Izquierdo et al., 2017).

Other common variables in most studies were a linear sprint (5, 10, 20 and 30 m) and a change of direction (COD). As for the linear sprint, the proposed distance was diverse, from a short 5-m sprint (Stojanović et al., 2021), a 10-m sprint (O Brien et al., 2020), the 20-m shuttle run test (Sánchez-Sánchez et al., 2019), a 25-m sprint with 5 and 20 m splits (Gonzalo-Skok et al., 2022) to a 30-m sprint (Cabanillas et al., 2020). The sprint test showed improvements after applying flywheel training at all the proposed distances, except the 20 m test applied by Stojanović et al. (2021) and the different splits that Gonzalo-Skok et al. (2022) used, achieving only a substantial improvement at 5 m. The former stated that the results did not show improvement possibly due to the training status of participants (inexperienced participants show more significant improvements than those with experience) or to the specificity of the test, suggesting that shorter speed tests such as of 5 or 10 m are more specific to basketball performance (Stojanović et al., 2021). On the other hand, following the results provided by Sánchez-Sánchez et al. (2019), an improvement could be seen in the 20 m shuttle run test for both groups, those who trained with flywheel and those who did not. In this case, it should be considered that this type of training was applied as a complement in a group where the main training methodology was based on high-intensity interval training (HIT).

Therefore, it is unlikely that the improvement in sprint times can be attributed solely to flywheel training, but rather to HIT carried out by both groups (Buchheit et al., 2010). Notably, despite both groups showing improvements compared to their respective baseline levels, there were no significant differences between them in the 20 m sprint test. Finally, if we look in-depth at the improvements obtained by O Brien et al. (2020) in the 10 m sprint test, we can associate them with the adaptation period after the season rest (since the study was carried out during the preseason with duration of 4 weeks) and perhaps not so closely linked to flywheel training, despite the fact that the group that trained iso-inertially obtained more significant improvements than the one that trained following a tempo-eccentric training methodology. In other sports such as soccer, although it is difficult to extrapolate it to basketball since it is an outdoor sport, benefits were also found in sprinting after applying iso-inertial training (de Hoyo et al., 2014; Fiorilli et al., 2020; Tous-Fajardo et al., 2016).

Regarding the change of direction, it was also postulated as a common evaluator for the different studies reviewed. All those who carried out this test obtained improvements in the flywheel group, except O Brien et al. (2020) who did not report improvements in the COD. The other authors did report improvements in the COD either using the T-test (Hernández-Davó et al., 2018; Stojanović et al., 2021), the Illinois test (Sánchez-Sánchez et al., 2019) or the COD 180º test (Gonzalo-Skok et al., 2022). Therefore, flywheel training appears to lead to improvements in COD times (Gonzalo-Skok et al., 2022; Hernández-Davó et al., 2018; Sánchez-Sánchez et al., 2019; Stojanović et al., 2021). Sánchez-Sánchez et al. (2019) stated that the improvements linked to their intervention were due to the strength increase obtained by participants who trained iso-inertially, since this is key in tasks such as the COD, and the group that carried out HIT only could not benefit from these increases in strength. It should be noted that Hernández-Davó et al. (2018), considering the improvements obtained in the COD, differentiated between the bilateral and the unilateral group (both trained iso-inertially), being the bilateral group the one that obtained the best results, which may be due, as the authors explained, to the similarity that the COD presents with the lower body when done bilaterally. In other sports such as soccer as well as in the CMJ, improvements were also obtained by implementing flywheel training (de Hoyo et al., 2014; Fiorilli et al., 2020; Gonzalo-Skok et al., 2016; Tous-Fajardo et al., 2016). In indoor sports such as handball, the effect of flywheel training on the COD has also been examined, highlighting the study by Madruga et al. (2020) which reported an improvement in COD performance in the group that trained using the conical pulley compared to the one who trained with traditional weights.

Finally, it should be mentioned that possible benefits associated with flywheel training also relate to prevention of injuries inherent to it. Gual et al. (2016) suggested that applying iso-inertial training to team sports players such as basketball and volleyball could improve lower body power without being associated with patellar tendon pain, thus linking iso-inertial training with injury prevention. Gonzalo-Skok et al. (2022), on the other hand, stated that flywheel training using a conical pulley, highlighting unilateral exercises, might help reduce inter-limb jumping asymmetries and therefore, minimize the risk of injury or be considered a tool to detect which players are at high risk of injury. Focusing on inter-limb asymmetries, Fort-Vanmeerhaeghe et al. (2022) in their recent study also suggested the use of flywheel methods (a conical pulley) in a battery of different tests to detect inter-limb asymmetry in female basketball players. Along the same line, we can find some studies that applied flywheel training and reduced the injury rate in participants, perhaps the most relevant were the findings of de Hoyo et al. (2014) who, after 10 weeks of flywheel training applied to youth elite soccer players, concluded that the eccentric resistance training program reduced the incidence and severity of muscle injuries in addition to improving variables such as the CMJ and the linear sprint. Also applied to elite soccer players, we found a study by Suarez-Arrones et al. (2020) where they applied eccentric overloading to lower body muscle groups in both the rotational pulley and the conical pulley, concluding, after measuring different muscle bellies and their activity by means of magnetic resonance, that the application of flywheel training could be beneficial to both prevent injuries and minimize the risk of relapse after return to play. These results clarify the theory that the use of eccentric overload training can prevent injuries (Petersen et al., 2011; Tesch et al., 2017) and suggest the use of flywheel training as a preventive method (Beato et al., 2021; Suarez-Arrones et al., 2020) as well as an effective method to improve performance (Maroto-Izquierdo et al., 2017; Tous-Fajardo et al., 2016).

## Conclusions

While flywheel training is not as common as other training protocols such as body weight training, it appears that this method shows benefits associated with basketball performance compared to traditional training methods. The present systematic review observed how this type of training applied to basketball players without previous experience in training with flywheel devices, could improve variables associated with performance such as the vertical jump, change of direction, and running speed. Few studies have applied this methodology in basketball players, thus future research should pay attention to this area to provide a more precise view of the improvements associated with flywheel training related to performance of basketball players.

## Practical Implications

Based on the results obtained from this systematic review, we believe that flywheel-based training, no less than 8 weeks, having previously performed a familiarization process with this technology, and with a volume of 4 sets of 8 repetitions, applied 1 or 2 days per week, for exercises focused on the lower limb such as a squat (bilateral or unilateral) or a Rumanian deadlift, may have performance-related benefits in basketball players.

## Future Guidelines

A future guideline would be to propose flywheel training intervention in more basketball teams, if possible professional, thus increasing the sample of studies to support the conclusions of this review. According to this line, we could also include increasing the number of players per study and also include the separation into two groups, i.e., a control group versus a flywheel training group, to increase the relevance or not of this type of training.

## Limitations and Strengths

One of the study's limitations is that participants were not blinded as were the personnel who carried out the training sessions. Participants were verbally encouraged by coaches to perform all the sets with maximum effort, an important extrinsic motivation factor for not being blinded. On the other hand, the number of studies could also be perceived as a limitation as there were only seven studies included for review. At the same time, this fact could be a strength as all the data related to the issue of basketball and flywheel, to the best of the author’s knowledge, has been summarized and examined. Last but not least, this review has a high-value level for basketball practitioners seeking to apply this methodology to improve basketball performance.
